# Characterizing Human Cell Types and Tissue Origin Using the Benford Law

**DOI:** 10.3390/cells8091004

**Published:** 2019-08-29

**Authors:** Sne Morag, Mali Salmon-Divon

**Affiliations:** Department of Molecular Biology, Faculty of Life Sciences, Ariel University, Ariel 40700, Israel

**Keywords:** single-cell RNA sequencing, Benford law, Benford distribution, cell classification, machine learning

## Abstract

Processing massive transcriptomic datasets in a meaningful manner requires novel, possibly interdisciplinary, approaches. One principle that can address this challenge is the Benford law (BL), which posits that the occurrence probability of a leading digit in a large numerical dataset decreases as its value increases. Here, we analyzed large single-cell and bulk RNA-seq datasets to test whether cell types and tissue origins can be differentiated based on the adherence of specific genes to the BL. Then, we used the Benford adherence scores of these genes as inputs to machine-learning algorithms and tested their separation accuracy. We found that genes selected based on their first-digit distributions can distinguish between cell types and tissue origins. Moreover, despite the simplicity of this novel feature-selection method, its separation accuracy is higher than that of the mean-expression level approach and is similar to that of the differential expression approach. Thus, the BL can be used to obtain biological insights from massive amounts of numerical genomics data—a capability that could be utilized in various biomedical applications, e.g., to resolve samples of unknown primary origin, identify possible sample contaminations, and provide insights into the molecular basis of cancer subtypes.

## 1. Introduction

The development of high-throughput genomic technologies, including microarray and next-generation sequencing (NGS), has led to the accumulation of massive amounts of transcriptomic data, creating new challenges for the research community; how can we handle the complexity of information stored in such massive datasets? How can this information be translated into a better understanding of basic biomedical mechanisms? And how should noisy data be handled so as to separate the wheat from the chaff? These are but a few questions that still require answers. Responding to these challenges requires novel algorithms and methods that can process ‘big data’ in a meaningful, accurate, robust, and computable manner. Solutions may come from the integration of principles from diverse fields, including mathematics, computer science, statistics, and physics; one such principle, which has only recently been described to have practical implications in the field of genomics, is the Benford law (BL; also known as the ‘first-digit law’).

An astonishing fact about the frequencies of first (leading) digits occurring in numerical data that describe natural phenomena is that the first digits are not evenly distributed, as might be expected; rather, they follow a logarithmic distribution, with 1 being the most prevalent first digit and 9 being the least prevalent. This unique phenomenon was originally described and theorized as a law by the astronomer Simon Newcomb in 1881 [1], and it was later noted again, in 1938, by Frank Benford [2], who tested it in numerous sets of physical constants and statistical data. This theorization was later known as the BL, which has been widely implemented in the detection of financial frauds [3,4]. However, the practical implications of the BL in other fields, including genomics, has emerged only recently [5,6,7,8,9,10,11,12].

Previous studies have revealed that leading digits of gene expression data follow the Benford distribution—both for microarray intensities [7] and for RNA-seq digital expression levels—in a manner that is stable across tissues and is robust to different normalizations [13]. Deviations from the Benford distribution were detected when different gene sets with unique characteristics were explored, demonstrating that the likelihood of tissue specificity can be predicted based on the Benford behavior of the genes in question [13]. Motivated by this finding, in the present study, we tested whether the adherence of gene sets to the Benford distribution can serve as a means by which to classify cells to their cell type and to detect the origin of a tissue sample.

Several methods and algorithms have been developed to classify cell types based on scRNA-seq data [14,15,16], all of which involve data dimensionality reduction followed by the clustering of cells into subgroups and the identification of genes that are expressed differentially between these clusters. The final step is the assignment of cell type labels to cell clusters, which requires that gene markers are available to allow an efficient and accurate discrimination between cell types. Most existing marker selection approaches are based on a differential expression (DE) analysis between a single cell type and all other cell types in a sample [17]. Due to the low coverage of high-throughput single-cell sequencing, it is preferable to use highly expressed markers, which are not always available. When the cell population is highly heterogeneous, shallow sequencing is sufficient to classify a cell type [18]; however, in more homogenous populations, deep sequencing may be required so as to improve the detection of genes with low expression [19], allowing the use of less abundant cell type markers. A marker-selection method that does not require any differential expression test or a comparison between cell types, and that is robust to expression variations between cells of the same type, may overcome these limitations. We suggest here that the BL can be used as such a feature selection approach.

If the BL is indeed efficient in such cases, it could be integrated into several biomedical applications that require tissue origin identification, cell-type separation, or cellular enumeration.

## 2. Materials and Methods

### 2.1. Datasets

The main scRNA-seq dataset used in this study was published by Chu et al. [20] and was obtained from GEO (accession number GSE75748). The data comprise six types of cells, which represent three levels of cell potency: high potency, which includes two types of pluripotent human embryonic stem cells (hESCs)—H1 and H9—that we combined to a single, “H1” group; medium potency (progenitors; multipotent), which includes neural progenitor cells (NPCs, ectoderm-derived), definitive endoderm progenitors (DEPs), endothelial cells (ECs, mesoderm-derived), and trophoblasts (TBs, extra-embryonically derived); and low potency (differentiated cells), which includes human foreskin fibroblasts (HFFs) (Table 1). To obtain raw counts, sequences were mapped to the human genome (hg38) using STAR [21], and the assignment of sequence reads to genes was conducted by using the featureCounts tool [22]. For tissue analysis, bulk RNA-seq data of 53 tissues (11,688 samples in total) (Table S1) were downloaded from the GTEx portal [23] using “Transcripts TPMs” version 7 together with its annotations dataset, “SampleAttributesDS”.

### 2.2. Analysis of Benford Distribution

**Figure 1:**
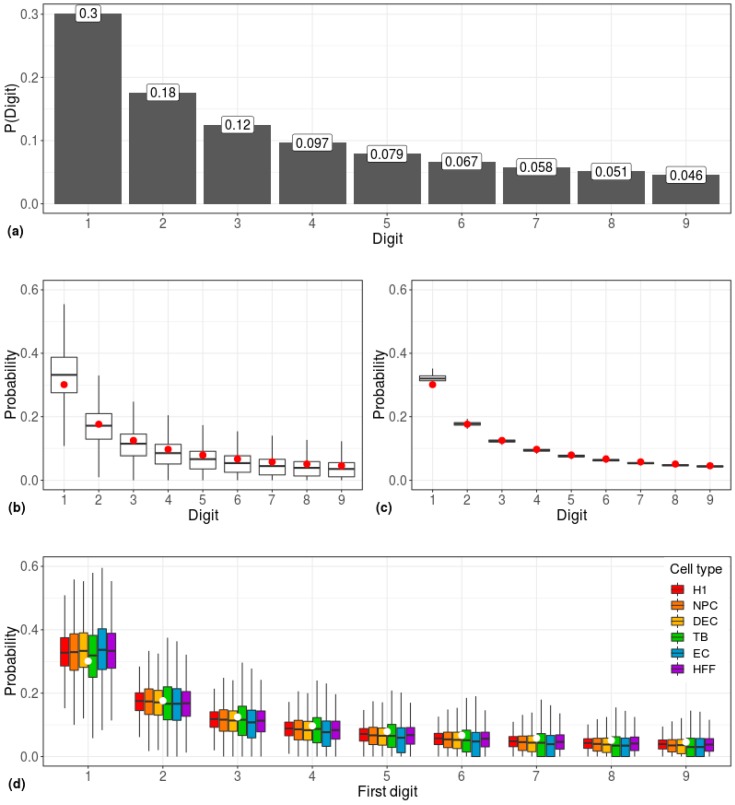
Correlations between scRNA-seq data and the Benford distribution. (**a**) Predicted prevalence of the occurrence of each leading digit according to the Benford Law. D = digit; P(Digit) = Frequency of D. (**b**) Gene-centered and (**c**) cell-centered first-digit distributions, calculated across 1,018 single cells. The predicted Benford distribution is represented by red dots. (**d**) First-digit distribution of gene-centered expression data of six cell types. White circles represent the predicted Benford distribution. H1: human embryonic stem cells (*n* = 375 cells); NPC: neural progenitor cells (*n* = 173 cells); DEP: definitive endoderm progenitors (*n* = 138 cells); EC: endothelial cells (*n* = 105 cells); TB: trophoblasts (*n* = 69 cells); HFF: human foreskin fibroblasts (*n* = 159 cells).

In a set of numbers found in real-world datasets, the BL posits that, rather than being distributed uniformly, lower digits occur as leading digits disproportionately more often than higher digits (Figure 1a).

An in silico analysis was conducted by using the R programming version 3.5.0 [24], the RStudio and additional R packages. The ‘BenfordTests’ [25] package (version 1.2.0) was used to calculate the Benford distributions of the different gene expression data. The mean absolute error (MAE), as defined in Equation (1) below, is the mean absolute deviation from the calculated (Ai) and expected (Ei) Benford distributions for all nine digits.

Equation (1): Mean absolute error (MAE) calculation.

(1) $$MAE=\frac{1}{9}\sum_{i=1}^{9}\left|Ai-Ei\right|$$

Both cell-centered and gene-centered MAE calculations were used. In the cell-centered calculation and in the Benford distribution analysis, the MAE scores were calculated across all genes in each cell. Conversely, in gene-centered calculations, the MAE scores were calculated separately for each gene in each group of cells, across all the cells within that certain group (Figure S1).

### 2.3. Lists of Genes

A list of pluripotency genes was obtained from Nathan et al. [26] and originally comprised 189 genes, associated with the pluripotency of stem cells. This list of genes was compared with the available list of genes from the dataset used here, totaling 178 genes that represent pluripotency. The MAE and the mean expression score (mean-EXP; see below) were calculated for each cell based on these genes.

Benford-based lists of genes were generated through a gene-centered calculation of the first-digit distribution and the MAE score for each gene in each cell type. This process was followed by selecting the 200 highest and lowest MAE genes, separately for each cell type, thus generating two gene-sets (high/low MAE genes), each containing six lists of genes (one list for each cell type). These six lists of genes were later used to calculate the cell-centered first-digit distribution and MAE for each cell in each of the six groups of cell types. The cell type from which the list of genes was extracted was termed the ‘focus group’.

Mean-EXP lists were generated by calculating the gene-centered mean-EXP for each gene across all cells of the same type and then selecting the 200 highest and lowest mean-EXP genes for each cell type. This process yielded two sets of genes (high-/low-expressed genes), each containing six lists of genes, based on which six cell-centered mean-EXP scores were generated for each cell.

To generate lists of differentially expressed (DE) genes, we used three approaches. First, we conducted *t*-tests to compare the expression of genes in the focus group with that in all other groups (cell types) combined. This process was repeated for each cell type so as to create six lists of genes, each comprising 200 most DE genes (*p* ≤ 0.05). Second, we used the Seurat R package (version 1.10.2) [27] to detect DE-based markers. Third, we used MAST [28], a GLM-framework that treats cellular detection rate as a covariate. Based on the six lists of DE genes (one list per cell type, generated by either approach), six polygenic scores (PS) were calculated for each cell. The PS of a cell was derived by computing the sum of the products of the gene coefficients (si; −1 or 1, depending on whether it is down- or upregulated, respectively) by the corresponding normalized gene expression value (ei), according to Equation (2) [29].

Equation (2): Polygenic score (PS) calculation.

(2) $$PS=\sum_{i=1}^{n}e_{i}*s_{i}$$

For MAE, low mean expression, and DE calculations, we used the median ratio normalization (MRN) data; for Seurat and MAST, we used the raw counts.

### 2.4. Statistical Analysis

To compare between the calculated and expected Benford distributions, Pearson’s Chi-square goodness-of-fit was used (R package BenfordTests [25]), where the null hypothesis supports the adherence of the data to the Benford Law (i.e., *p* > 0.05 supports the adherence to the Benford distribution). To compare the distribution of MAE and Mean-EXP values of each cell type to that of the other cell types combined (Figure 3, Figure 4 and Figure 5), we used the Wilcoxon test.

### 2.5. Clustering and Machine Learning

We examined the ability of a computer to learn and identify cell types based on their Benford distributions. Since each gene set comprised six sub-lists (one for each of the six cell types), each cell was eventually represented by six MAE, mean-EXP, and PS scores. These scores, obtained for each cell individually, were used for t-distributed stochastic neighbor embedding (tSNE) visualization using Rtsne (version 0.15) [30] and for a principle component analysis (PCA) clustering and machine-learning prediction, by using the Caret (version 6.084) [31] R package.

The dataset was divided into a training set, containing 80% of the cells, and a test set, containing the remaining 20% of the cells. The desired gene sets (high-/low-MAE, mean-EXP, or DE) were acquired based on the training set for cell-centered calculations to yield six values for each cell. These cell-centered values were applied in five algorithms: random forest (RF), linear, and radial support-vector machine (SVM), partition around medoids (PAM), and linear discriminant analysis (LDA). A 10-fold cross-validation was performed based on the training dataset to detect the best set of algorithmic parameters, and then the model was tested on the test sets’ cell-centered six-scores (calculated based on the gene-sets acquired from the training data). The finally selected parameter settings for classification are shown in Table S2. Prediction evaluation was conducted using the area under the ROC curve (AUC), which represents the true-positive prediction results. Multiclass AUC was calculated using the pROC R package (version 1.15.0) [32]. This procedure was repeated 10 times, each time using a different 80% and 20% of the data of Chu et al. [20].

GTEx [23] analysis was conducted in a similar manner, on 53 tissues (i.e., each gene set contained 53 gene lists, eventually leading to 53 scores that represent each sample), but using the Radial-SVM, RF, and LDA algorithms. Due to the large size of the dataset, the 80%/20% division of the data used to train and test the algorithm was repeated once. “Cervix Endocervix” tissue samples were excluded from DE analyses because they included only five samples (Table S1).

## 3. Results

The first-digit distribution of gene expression values, obtained from 1,018 single cells belonging to six cell types, adhered to the Benford distribution (Figure 1). This phenomenon was observed both when employing a gene-centered approach (calculating the distribution for each gene individually; Figure 1b) and when employing a cell-centered approach (i.e., calculating the distribution for each cell individually; Figure 1c), and it was irrespective of the type of cell (Figure 1d). While the Pearson Chi-square goodness-of-fit test rejected the hypothesis of data conformity to the Benford distribution (*p* < 10–16), possibly due to minor deviations within the first-digit frequencies, the Benford-like distribution is clear.

**Figure 2:**
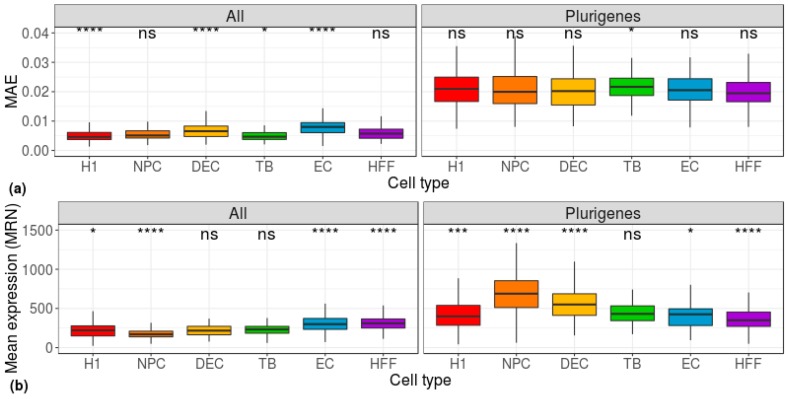
Distribution of group-based, cell-centered (**a**) MAE and (**b**) mean-expression scores, calculated across all genes (left) and across 178 pluripotent genes (right) [26]. H1: human embryonic stem cells (*n* = 375 cells); NPC: neural progenitor cells (*n* = 173 cells); DEP: definitive endoderm progenitors (*n* = 138 cells); EC: endothelial cells (n = 105 cells); TB: trophoblasts (*n* = 69 cells); HFF: human foreskin fibroblasts (*n* = 159 cells). Each group was compared to all other cell types, combined, using a Wilcoxon test. Ns: non-significant (*p* > 0.05), *p ≤ 0.05, **p ≤ 0.01, ***p ≤ 0.001, ****p ≤ 0.0001.

Next, since our data comprised cells of three levels of potency (pluripotent, multipotent, and differentiated), we used the known pluripotency signatures of the genes [26] as our dataset. The distribution of the MAE, calculated for each individual cell across the pluripotency genes (i.e., using a cell-centered approach), was similar for all cell types (Figure 2a). Using the mean-EXP values to differentiate between the cell types did not yield much better separation (Figure 2b).

**Figure 3:**
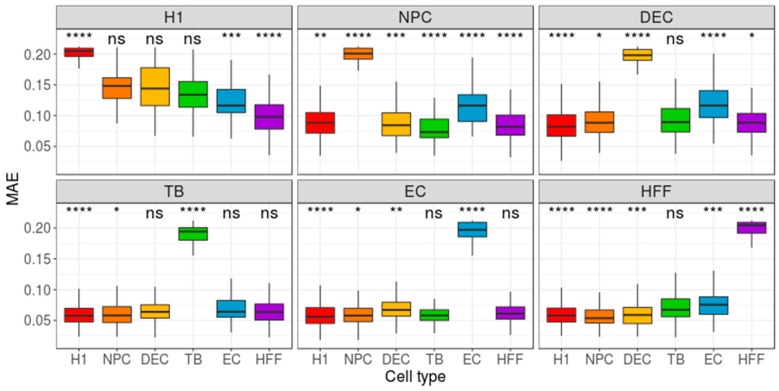
Distribution of group-based, cell-centered MAE scores, calculated across 200 genes that have the highest gene-centered MAE scores. The 200 genes with the highest MAE scores were detected separately for each cell type, and then the cell-centered MAE score was calculated based on these genes. Panel titles indicate the focus groups (for example, the panel entitled ‘H1’ shows the cell-centered MAE score distribution, calculated for each cell type, based on 200 genes that have the highest gene-centered MAE scores within the H1 group). H1: human embryonic stem cells (*n* = 375 cells); NPC: neural progenitor cells (*n* = 173 cells); DEP: definitive endoderm progenitors (*n* = 138 cells); EC: endothelial cells (*n* = 105 cells); TB: trophoblasts (*n* = 69 cells); HFF: human foreskin fibroblasts (*n* = 159 cells). Each group was compared to all other cell types, combined, using a Wilcoxon test. ns: non-significant (*p* > 0.05), *p ≤ 0.05, **p ≤ 0.01, ***p ≤ 0.001, ****p ≤ 0.0001.

**Figure 4:**
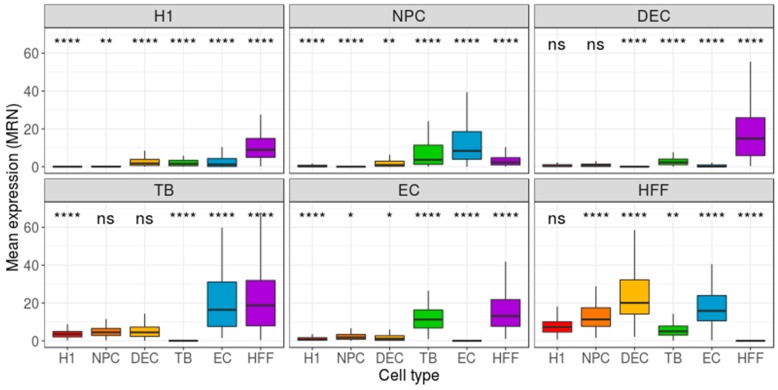
Distribution of group-based, cell-centered mean-expression (mean-EXP) scores, calculated across the 200 genes that were found to have the lowest gene-centered mean-EXP scores. The 200 genes with the lowest mean-EXP score values were detected separately for each cell type, and then cell-centered mean-EXP scores were calculated, based on these genes. Panel titles indicate the focus groups. H1: human embryonic stem cells (*n* = 375 cells); NPC: neural progenitor cells (*n* = 173 cells); DEP: definitive endoderm progenitors (*n* = 138 cells); EC: endothelial cells (*n* = 105 cells); TB: trophoblasts (*n* = 69 cells); HFF: human foreskin fibroblasts (*n* = 159 cells). Each group was compared to all other cell types, combined, using a Wilcoxon test. Ns: non-significant (*p* > 0.05), *p ≤ 0.05, **p ≤ 0.01, ***p ≤ 0.001, ****p ≤ 0.0001.

**Figure 5:**
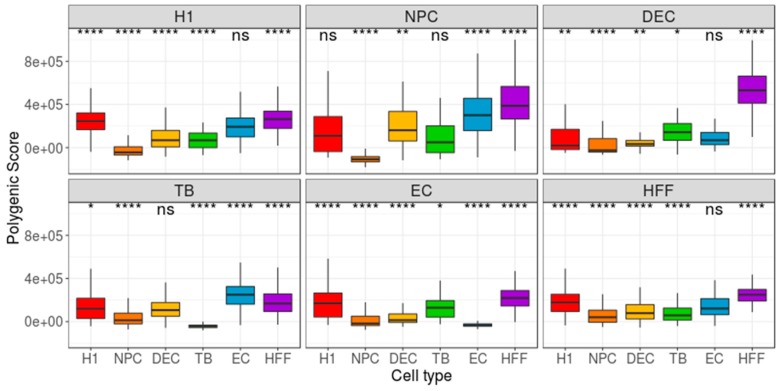
Distribution of group-based, cell-centered polygenic scores (PS), calculated across the 200 most differentially expressed (DE) genes. The 200 DE genes were detected separately for each cell type, and then cell-centered PS scores were calculated, based on these genes. Panel titles indicate the focus group. H1: human embryonic stem cells (*n* = 375 cells); NPC: neural progenitor cells (*n* = 173 cells); DEP: definitive endoderm progenitors (*n* = 138 cells); EC: endothelial cells (*n* = 105 cells); TB: trophoblasts (*n* = 69 cells); HFF: human foreskin fibroblasts (*n* = 159 cells). Each group was compared to all other cell types, combined, using a Wilcoxon test. Ns: non-significant (*p* > 0.05), *p ≤ 0.05, **p ≤ 0.01, ***p ≤ 0.001, ****p ≤ 0.0001.

As the Benford distribution of a known pluripotency set of genes was unable to distinguish between the different types of cells, we next extracted, for each cell type, two sets of genes, each comprising 200 genes that have either the highest or lowest gene-centered MAE value. Then, we calculated the MAE value of each cell (cell-centered MAE), based on the first-digit frequency of the genes included in the selected gene-set (high/low MAE). If each cell type is characterized by a unique set of genes with a high (or low) MAE score, one could expect the distribution of MAE scores, calculated for this set of genes, to vary between different cell types. While the lowest MAE-gene signature was unable to distinguish between the types of cells (Figure S2), the highest MAE-gene signature yielded a significant separation between the focus group and the other cell types (p < 0.0001, Figure 3). Repeating this pipeline using mean-EXP (instead of MAE) for feature selection revealed that, while the average expression distribution calculated based on the 200 lowest mean-EXP genes was significantly different between the focus group and the other cell types (*p* < 0.0001, Figure 4), the group separation was not as robust as the separation based on the Benford-based analysis. For example, the mean expression distribution of the 200 lowly expressed genes in H1 cells was similar to that of the same genes in NPCs (Figure 4, top left panel). Using the mean expression of the 200 highest mean-EXP genes yielded a poorer separation (Figure S3). Since feature-selection methods are usually based on the DE of genes—rather than choosing genes based on their levels of expression—we sought to compare the performance of our Benford-based algorithm to that of cell-type separation based on the analysis of DE genes. To this end, we selected genes that are DE between the focus group and all other cell types and used these genes for cell-centered PS calculations (see Methods). Although the distribution of the PS values was significantly different between the cell types (p < 0.0001), the distributions of different cell types overlapped without clear separation (DE: Figure 5; Seurat’s genes: Figure S4; MAST genes: Figure S5). For instance, the distribution of PS values calculated based on H1 DE genes was similar in both H1 cells and HFF (Figure 4, top left panel).

**Figure 6:**
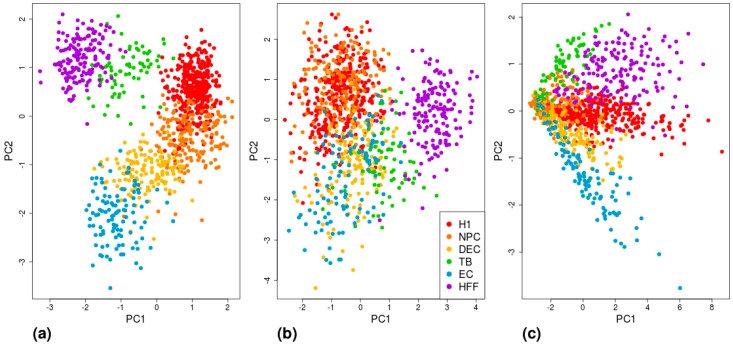
Visualization of cell-type separation using PCA. Each cell is represented by (**a**) six MAE values, calculated based on the 200 high-MAE genes of each cell type; (**b**) six expression values, averaged across the 200 lowest expressed genes for each cell type; or (**c**) six polygenic scores, calculated based on 200 DE genes of each cell type. H1: human embryonic stem cells (*n* = 375 cells); NPC: neural progenitor cells (*n* = 173 cells); DEP: definitive endoderm progenitors (*n* = 138 cells); EC: endothelial cells (*n* = 105 cells); TB: trophoblasts (*n* = 69 cells); HFF: human foreskin fibroblasts (*n* = 159 cells).

The sets of highest MAE genes, low mean-EXP genes, and DE genes for each cell type according to the three methods are shown in Table S3. To further visualize and compare the efficiency of the different gene sets in differentiating between cell types, we conducted a tSNE analysis (Figure S7) and a PCA (Figure 6; and Figure S6 for the DE results based on Seurat and MAST), which revealed that the most successful clustering by cell type was achieved by using the Benford-based (high MAE) algorithm, followed by the DE gene analysis.

**Figure 7:**
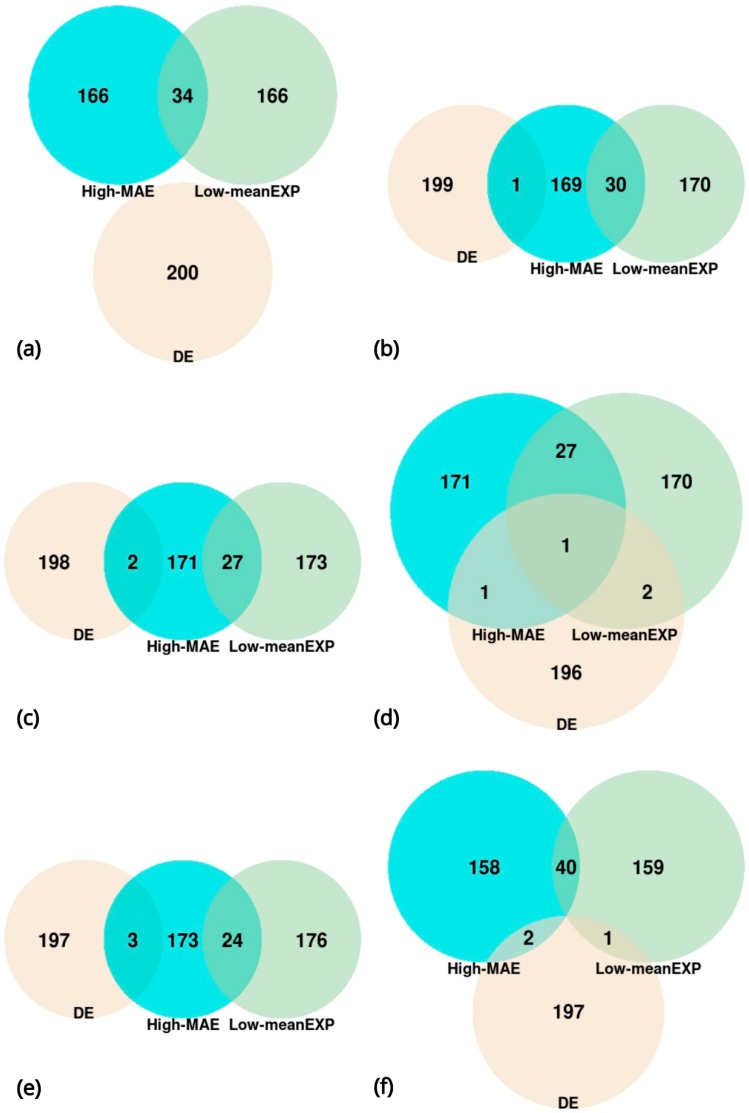
Overlap between the 200 high-MAE genes, the 200 low mean-expression (mean-EXP) genes, and 200 differentially expressed (DE) genes of each cell type. (**a**) Human embryonic stem cells list (H1, *n* = 375 cells); (**b**) neural progenitor cells list (NPC, *n* = 173 cells); (**c**) definitive endoderm progenitors list (DEC, *n* = 138 cells); (**d**) trophoblasts list (TB, *n* = 69 cells); (**e**) endothelial cells list (EC, *n* = 105 cells), and human foreskin fibroblasts list (HFF, *n* = 159 cells).

**Figure 8:**
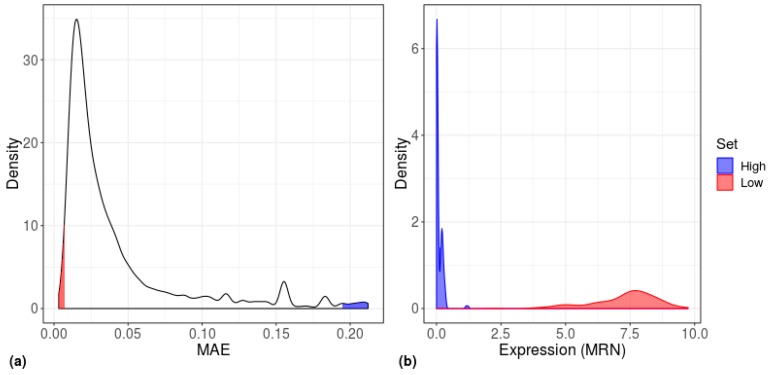
Distribution of gene-centered MAE scores for human embryonic stem cells (H1). (**a**) Density plot of MAE scores, calculated separately for each gene (*n* = 19,097 genes) across all H1 cells (*n* = 375 cells). (**b**) Density plot of the expression values of the highest (blue) and lowest (red) MAE genes of the H1 cells.

Since the gene sets that led to a significant separation comprised the 200 high-MAE, low mean-EXP, and DE genes, we next examined the overlap between these gene sets. In all types of cells, the greatest overlap was between high-MAE genes and low mean-EXP genes, whereas all other groups showed almost no overlap (Figure 7). The relatively high overlap between high-MAE and low mean-EXP genes prompted us to explore the expression level distribution of the 200 highest and 200 lowest MAE genes (Figure 8); this analysis revealed that low-MAE genes have a wide expression distribution and are highly expressed, while high-MAE genes have a narrow expression distribution and are lowly expressed, as have been shown previously [13].

**Figure 9:**
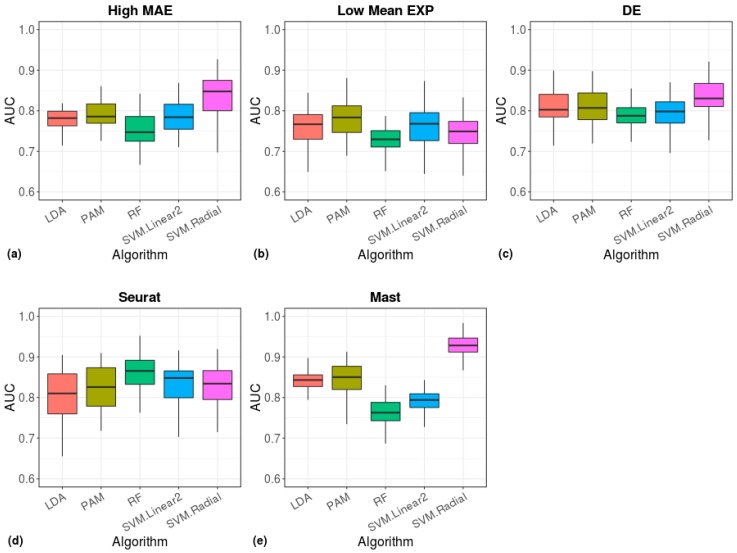
Cell type prediction accuracy of machine-learning algorithms. The area under the ROC curve (AUC), showing the performance of the indicated models in classification of cell types. The models were based on cell scores calculated from the 200 high-MAE (**a**), low mean-EXP (**b**), DE (**c**), Seurat (**d**), or MAST (**e**) gene sets.

To quantify the effectiveness of the Benford-based algorithm in clustering cell types, we ran five machine learning algorithms and evaluated the classification performance using the measurement of AUC (Figure 9, Table S4). The radial-SVM algorithm yielded the highest accuracy (median AUC ≈ 0.85, Figure 9a), which was similar to the accuracy of a model based on DE genes (Figure 9c). A similar accuracy was achieved by Seurat (RF median AUC ≈ 0.86) and a higher accuracy was achieved by MAST (radial-SVM median AUC ≈ 0.93).

**Figure 10:**
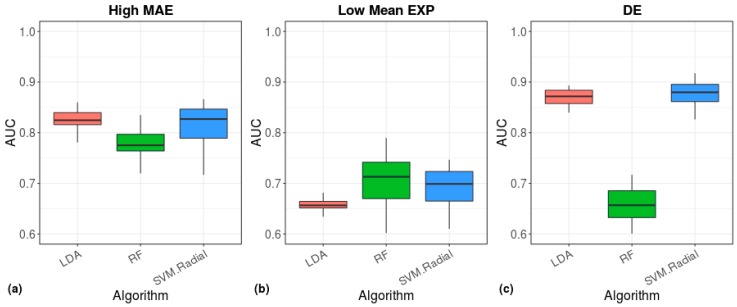
Tissue prediction accuracy of machine-learning algorithms. The area under the ROC curve (AUC), showing the performance of the indicated models in classification of tissue origin. The models were based on cell scores calculated from the 200 high-MAE (**a**), low mean-EXP (**b**), or the DE (**c**) gene sets.

The above-mentioned findings demonstrate the ability of calculations based on the BL to accurately separate cell types by using single-cell sequencing data. Since each tissue comprises many types of cells, we next tested the ability of our Benford algorithm to distinguish between different tissues and to detect the origin of a given tissue sample. We previously found, for 16 tissues that are included in the Illumina Human BodyMap 2.0 dataset (measured using microarray technology), that bulk gene expression data follow the Benford distribution when all genes are included in the calculation [13]. Therefore, we repeated this analysis, this time using GTEx bulk RNA data from 53 different tissues; similar to our previous results, we found that the first-digit distribution of the tissues adheres to the Benford distribution (Figure S8). Next, for each tissue included in the GTEx dataset, we detected a set of high-MAE, low mean-EXP, and DE genes and used them as inputs to machine-learning algorithms to evaluate their tissue classification performance (Figure 10). The radial-SVM and LDA algorithms demonstrated the highest accuracy for DE genes (median AUC ≈ 0.88, Figure 10c) and for the highest MAE genes (AUC ≈ 0.84, Figure 10a), whereas low mean-EXP genes demonstrated a lower accuracy (AUC ≈ 0.7 and 0.81 for the GTEx dataset and the dataset of Chu et al., respectively), which was mostly represented by the RF method (Figure 10b).

## 4. Discussion

The development of high-throughput genomic technologies and the reduction of their cost resulted in very large datasets, allowing the application of the BL in the field of genomics. The observation that general gene expression data follow the BL suggests that deviation from the Benford distribution of specific genes may be a characteristic of cell type or tissue origin. Our results support this hypothesis, as we found that both cells and tissues can be classified using information on the first-digit frequencies of sets of genes. Unexpectedly, accurate classification was observed not by the most Benford-adherent genes but by the most Benford-deviant genes, which are characterized by poor expression levels. One possible explanation for this phenomenon is the narrow range of expression of these genes; whereas numerical data that adhere to the Benford distribution span multiple orders of magnitude [33]. The selected Benford-deviant genes showed a narrow expression distribution, meaning that their expression across cells of the same type is restricted, thus allowing their accurate clustering. Nevertheless, the dependency of the algorithm on poorly expressed genes may be a limitation due to the over-abundances of zero-values in single-cell data. Hence, the ability of the BL-based method to deal with zero inflation should be investigated in future studies.

Unlike single-cell classification approaches that are based on the expressional magnitude [34], differential expression [35], or biological characteristics of the genes that directly affect their expression [36], classification according to adherence to the BL does not rely on gene expression level. Rather, it is based on the stochastic nature of first-digit occurrence probabilities, which potentially makes Benford-based classification more robust to fluctuations and variations in gene expression between cells of the same type or between tissues of the same origin. In addition, feature selection methods that are often used to classify cells according to their type rely on DE and require a statistical comparison of gene expression between the cell types, which, in many cases, is underpowered due to the large number of genes that are simultaneously analyzed [37], or when many conditions are being compared. For example, Seurat [27], in its default behavior, uses the Wilcoxon Rank Sum test to identify differentially expressed genes between two groups of cells, while MAST [28] uses a hurdle model tailored to scRNA-seq data. In contrast to the DE-based approach, a Benford-based analysis does not require statistical testing and is much simpler and more straightforward, requiring only the Benford adherence of genes for feature selection. Notwithstanding its simplicity, we found that the cell-separation accuracy of this approach is comparable to that of the DE approach and is better than using only expression levels (i.e., the mean-EXP approach). Unlike typical single-cell classification pipelines, which require initial clustering of cells prior to classification [14,38], the BL approach does not require the clustering of cells. Each cell is characterized by its Benford behavior of sets of genes, and this information is directly used in a machine-learning step to enable their classification. Hypothetically, if we knew the gene expression levels in all possible cell types, we could classify an individual cell to its type simply by implementing our method on its transcriptome. Until this occurs, our approach can be used to characterize the type of each individual cell in a mixture of cells of known types. To conclude, our findings implicate the BL as a novel, simple, and accurate method for feature selection, which, in the field of genomics, can be employed for defining cell types, tissues, and differentiation potencies using either single-cell or bulk RNA-seq data. In light of the recent advancements in methods for single-cell transcriptomic sequencing of various cell types, we believe that the BL algorithm can be further developed to define all different cell types, so as to be further integrated in various classification pipelines.

## Figures and Tables

**Table 1:** The dataset from Chu et al. [20], used in this study, indicating the number of single cells within each cell type.

Cell Type	Potency	Number of Cells
Human embryonic stem cells (hESC)	Pluripotent	374
Neural progenitor cells (NPC)	Multipotent	173
Definitive endoderm progenitors (DEP)		138
Endothelial cells (EC)		105
Trophoblasts (TB)		69
Human foreskin fibroblasts (HFF)	Differentiated	159

## Supplementary Materials

The following are available online at https://www.mdpi.com/2073-4409/8/9/1004/s1. Table S1: The GTEx database (v7) used in this study, indicating the number of samples within each tissue; Table S2: The finally selected parameter settings for classification models; Table S3: The 200 highest MAE genes, low mean-EXP, and DE genes, as detected for each cell type; Table S4: Multiclass AUC for the different machine-learning models; Figure S1: Gene- and cell-centered MAE calculations; Figure S2: Distribution of group-based, cell-centered MAE scores calculated across the 200 genes that were found to have the lowest gene-centered MAE scores; Figure S3: Distribution of group-based, cell-centered mean-expression (mean-EXP) scores, calculated across the 200 genes that were found to have the highest gene-centered mean-EXP scores; Figure S4: Distribution of group-based, cell-centered polygenic scores (PS), calculated across the top 200 marker genes as detected by the Seurat package; Figure S5: Distribution of group-based, cell-centered polygenic scores (PS), calculated across the top 200 marker genes, as detected by MAST; Figure S6: Visualization of cell-type clustering separation using principle component analysis upon six polygenic scores, calculated based on 200 Seurat and MAST DE genes of each cell type; Figure S7: Visualization of cell-type clustering separation using tSNE analysis; Figure S8: First-digit distribution of gene-centered expression data for the 53 tissues included in the GTEx dataset.
