# Supplementary material for: Characterizing Human Cell Types and Tissue Origin Using the Benford Law
Source: Cells. 2019 Aug 29;8(9):1004. doi: 10.3390/cells8091004 (PMC6770594; doi:10.3390/cells8091004)
Supplement: Supplementary file 1 [file cells-08-01004-s001.zip › cells-542672Supp.pdf]

Supplementary material

Table S1: The GTEx [1] **database (v7) used in this study,** indicating the number of samples within each tissue.

Tissue	Number of Samples	Tissue	Number of Samples
Adipose, subcutaneous	442	Colon, sigmoid	233
Adipose, visceral Omentum	355	Colon, transverse	274
Adrenal gland	190	Esophagus, gastroesophageal junction	244
Artery, aorta	299	Esophagus, mucosa	407
Artery, coronary	173	Esophagus, muscularis	370
Artery, tibial	441	Fallopian tube	7
Bladder	11	Heart, atrial appendage	297
Brain, amygdala	100	Heart, left ventricle	303
Brain, anterior cingulate cortex BA24	121	Kidney, cortex	45
Brain, caudate basal ganglia	160	Liver	175
Brain, cerebellar Hemisphere	136	Lung	427
Brain, cerebellum	173	Minor salivary gland	97
Brain, cortex	158	Muscle, skeletal	564
Brain, frontal Cortex BA9	129	Nerve, tibial	414
Brain, hippocampus	123	Ovary	133
Brain, hypothalamus	121	Pancreas	248
Brain, nucleus accumbens basal ganglia	147	Pituitary	183
Brain, putamen basal ganglia	124	Prostate	152
Brain, spinal cord cervical c1	91	Skin, not sun-exposed, suprapubic	387
Brain, substantia nigra	88	Skin, sun-exposed, lower leg	473
Breast, mammary tissue	290	Small intestine, terminal Ileum	137
Cells, EBV-transformed lymphocytes	130	Spleen	162
Cells, transformed fibroblasts	343	Stomach	262
Cervix, ectocervix	6	Testis	259
Cervix, endocervix	5	Thyroid	446
Vagina	115	Uterus	111

Whole blood	407	

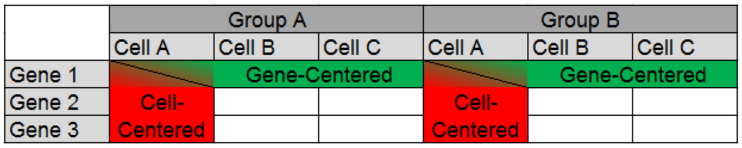

Figure S1: Gene- and cell-centered MAE calculations.

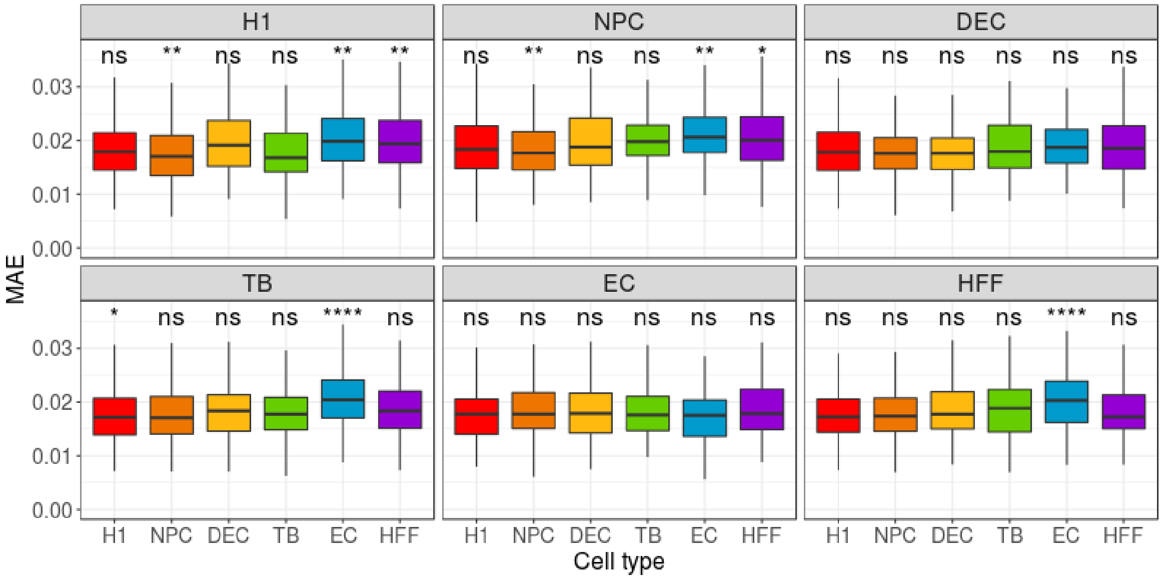

Figure S2: Distribution of group-based, cell-centered MAE scores calculated across the 200 genes that were found to have the lowest gene-centered MAE scores. The 200 genes with the lowest MAE scores were detected separately for each cell type, and then cell-centered MAE scores were calculated based on these genes. Panel titles represent the focus group (e.g., the "H1" panel indicates the cell-centered MAE score distribution calculated for each cell type, based on the 200 genes that have the lowest gene-centered MAE scores within the H1 group). H1: human embryonic stem cells (n = 375 cells), NPC: neural progenitor cells (n = 173 cells), DEP: definitive endoderm progenitors (n = 138 cells), EC: endothelial cells (n = 105 cells), TB: trophoblasts (n = 69 cells), HFF: human foreskin fibroblasts (n = 159 cells). Each group was compared with each of the other groups using a Wilcoxon test. Ns: non-significant ($p > 0.05$), *$p \leq 0.05$, **$p \leq 0.01$, ***$p \leq 0.001$, ****$p \leq 0.0001$.

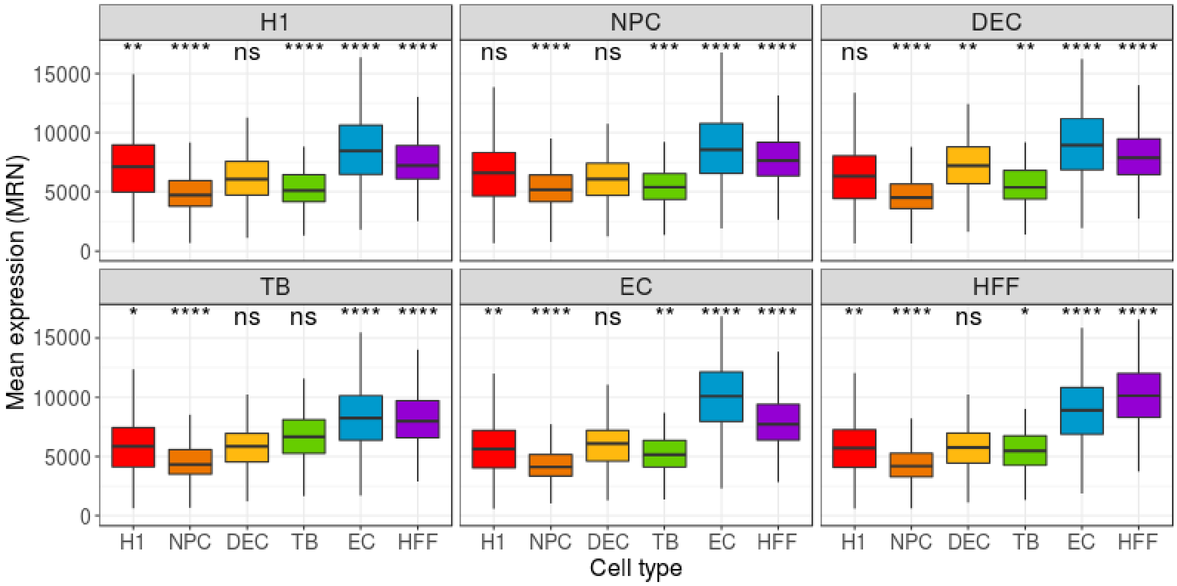

Figure S3: Distribution of group-based, cell-centered mean-expression (mean-EXP) scores, calculated across the 200 genes that were found to have the highest gene-centered mean-EXP scores. The 200 genes with the highest mean-EXP scores were detected separately for each cell type, and then cell-centered mean-EXP scores were calculated, based on these genes. Panel titles indicate the focus group. H1: human embryonic stem cells (n = 375 cells), NPC: neural progenitor cells (n = 173 cells), DEP: definitive endoderm progenitors (n = 138 cells), EC: endothelial cells (n = 105 cells), TB: trophoblasts (n = 69 cells), HFF: human foreskin fibroblasts (n = 159 cells). Each group was compared with each of the other groups using a Wilcoxon test. Ns: non-significant (p > 0.05), *p ≤ 0.05, **p ≤ 0.01, ***p ≤ 0.001, ****p ≤ 0.0001.

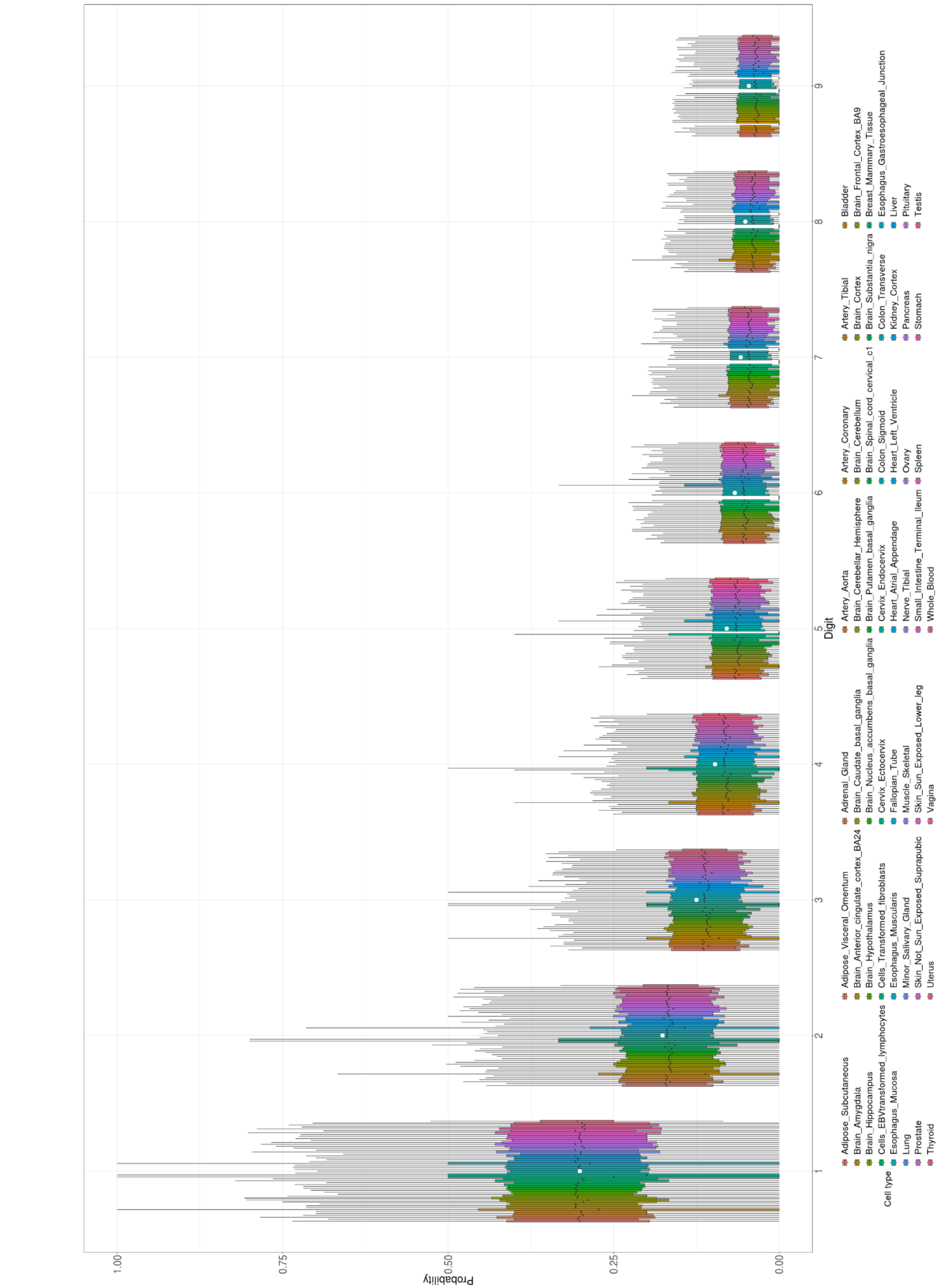
Probability
1.00
0.75
0.50
0.25
0.00
Digit
1
2
3
4
5
6
7
8
9
Cell type
Adipose_Subcutaneous
Adipose_Visceral_Omentum
Adrenal_Gland
Artery_Aorta
Artery_Coronary
Artery_Tibial
Bladder
Brain_Amygdala
Brain_Anterior_cingulate_cortex_BA24
Brain_Caudate_basal_ganglia
Brain_Cerebellar_Hemisphere
Brain_Cerebellum
Brain_Cortex
Brain_Frontal_Cortex_BA9
Brain_Hippocampus
Brain_Hypothalamus
Brain_Nucleus_accumbens_basal_ganglia
Brain_Putamen_basal_ganglia
Brain_Spinal_cord_cervical_c1
Brain_Substantia_nigra
Breast_Mammary_Tissue
Cells_EBVtransformed_lymphocytes
Cells_Transformed_fibroblasts
Cervix_Ectocervix
Cervix_Endocervix
Colon_Sigmoid
Colon_Transverse
Esophagus_Gastroesophageal_Junction
Esophagus_Mucosa
Esophagus_Muscularis
Fallopian_Tube
Heart_Atrial_Appendage
Heart_Left_Ventricle
Kidney_Cortex
Liver
Lung
Minor_Salivary_Gland
Muscle_Skeletal
Nerve_Tibial
Ovary
Pancreas
Pituitary
Prostate
Skin_Not_Sun_Exposed_Suprapubic
Skin_Sun_Exposed_Lower_leg
Small_Intestine_Terminal_Ileum
Spleen
Stomach
Testis
Thyroid
Uterus
Vagina
Whole_Blood

Figure S4: First-digit distribution of gene-centered expression data for 53 tissues included in the GTEx dataset [1]**.** White circles represent the Benford first-digit distribution. See **Table S2** for further details.

References

1. GTEx Portal Available online: https://gtexportal.org/home/.
